# The relationship between homeworking during COVID-19 and both, mental health, and productivity: a systematic review

**DOI:** 10.1186/s40359-023-01221-3

**Published:** 2023-06-27

**Authors:** Charlotte E. Hall, Louise Davidson, Samantha K. Brooks, Neil Greenberg, Dale Weston

**Affiliations:** 1 Department of Psychological Medicine, King’s College London, Weston Education Centre, London, SE5 9RJ UK; 2 Evaluation & Translation Directorate, Science Group, Behavioural Science and Insights Unit, UKHSA, Porton Down, Salisbury, SP4 0JG UK; 3 Health Protection Research Unit, Institute of Psychology, Psychiatry and Neuroscience, King’s College London, 10 Cutcombe Road, London, SE5 9RJ UK; 4 School of Psychology, University of Sussex, Brighton, BN1 9QH UK

**Keywords:** Work from home, WFH, Resilience, Mental health, Productivity, Review

## Abstract

**Background:**

As of March 2020, the UK public were instructed to work from home where possible and as a result, nearly half of those in employment did so during the following month. Pre-pandemic, around 5% of workers chose to work from home; it was often seen as advantageous, for example due to eliminating commuting time and increasing flexibility. However, homeworking also had negative connotations, for example, blurred boundaries between work and home life due to a sense of constant connectivity to the workplace. Understanding the psychological impact of working from home in an enforced and prolonged manner due to the COVID-19 pandemic is important. Therefore, this review sought to establish the relationship between working from home, mental health, and productivity.

**Methods:**

In January 2022, literature searches were conducted across four electronic databases: Medline, Embase, PsycInfo and Web of Science. In February 2022 grey literature searches were conducted using Google Advanced Search, NHS Evidence; Gov.uk Publications and the British Library directory of online doctoral theses. Published and unpublished literature which collected data after March 2020, included participants who experienced working from home for at least some of their working hours, and detailed the association in terms of mental health or productivity were included.

**Results:**

In total 6,906 citations were screened and 25 papers from electronic databases were included. Grey literature searching resulted in two additional papers. Therefore, 27 studies were included in this review. Findings suggest the association between homeworking and both, mental health and productivity varies considerably, suggesting a complex relationship, with many factors (e.g., demographics, occupation) having an influence on the relationship.

**Conclusion:**

We found that there was no clear consensus as to the association between working from home and mental health or productivity. However, there are indications that those who start homeworking for the first time during a pandemic are at risk of poor productivity, as are those who experience poor mental health. Suggestions for future research are suggested.

**Supplementary Information:**

The online version contains supplementary material available at 10.1186/s40359-023-01221-3.

## Background

Within the UK, the COVID-19 pandemic led to several behavioural interventions being implemented by the government with the aim to reduce transmission of the virus. As of March 2020, the public were instructed to work from home and as a result, nearly half of those in employment did so during April 2020 [1]. As of January 2022, 36% of workers still reported homeworking at least once in the last seven days [2]. Pre-pandemic, only around 5% of workers chose to work from home [3] and findings on the impact of doing so is inconsistent. For some, homeworking was seen as a positive way of overcoming issues (e.g., decreasing commuting time [4]). However, homeworking also had negative connotations, for example, blurred boundaries between work and home life due to a sense of constant connectivity to the workplace [5]. Considering the potential disadvantages of homeworking pre-pandemic, understanding the psychological effect of enforced and prolonged working from home due to the COVID-19 pandemic is important.

Unsurprisingly, since the onset of the pandemic, the association between working from home and various aspects of health have been the subject of much research. Literature reviews, including papers from pre-pandemic, have reported mixed findings. For example, a rapid review conducted by Oakman (2020), contained 23 studies published between 2008 and 2020, explored the link between working from home and mental and physical health. For mental health specifically, the relationship was reported to be complex with many conflicting findings (e.g., increased stress and increased well-being; [6]). Varied findings have also been reported by a systematic review conducted by Lunde (2022) which sought to establish the relationship between working from home and employee health (examined outcomes included: general health, pain, well-being, stress, exhaustion and burnout, satisfaction, life and leisure) using studies published between 2010 to 2020 [7].

A scoping review focused on more current pandemic related research was conducted by Elbaz (2022) and aimed to establish the association between telework (i.e., a working arrangement that allows individuals to engage in work activities through information and communication technologies from outside the main work location [8]) and work-life balance using studies published between January 2020 and December 2021. 42 papers were included, and the review concluded that teleworking resulted in a mixed relationship. However, the link between teleworking and psychological health was typically more negative than positive [8].

Thus, the purpose of this review is to establish if there is an association between working from home and both, mental health, and productivity; specifically, for those who experienced working from home during the COVID-19 pandemic. This systematic review seeks to, first, contribute to the evidence base by being the first review to collate findings from published and grey literature research originating from economically developed countries (as indicated by membership of the Organisation for Economic Co-operation and Development; OECD) into the link between working from home and both, mental health, and productivity during the COVID-19 pandemic. Second, to establish risk or resilience (as defined as positive adaptation in response to adversity [9]) factors that make an individual more likely to adapt well to homeworking during a pandemic. Third, to provide findings and conclusions that can be used to establish implications and future research suggestions for improving the experience of homeworking for those doing so during a future public health emergency.

## Method

This systematic review is designed in concordance with the Preferred Reporting Items for Systematic Reviews and Meta-Analyses (PRISMA) guidelines [10]. This results in the method section describing and explaining the process of criteria selection, use of information sources, the search strategy, study selection, data collection, quality assessment and the analytical method used during the review.

### Eligibility criteria

The development of inclusion and exclusion criteria for the current review was iterative and developed alongside literature familiarisation, preliminary database searches, and research team meetings. The final inclusion and exclusion criteria for the current systematic review can be found in Table 1.

**Table 1:** Inclusion and exclusion criteria

Category	Inclusion	Exclusion
**Type of Study**	Published and unpublished unique research (e.g., governmental reports, non-governmental reports, or graduate or undergraduate thesis or dissertation)^1^	Non-primary research (e.g., reviews, commentaries)
	Data was collected after March 2020	Data was collected pre-March 2020
	Published in English	Not available in English
	Full text available	Full text not available
	The research must have been conducted in an OECD country	The research was conducted in a non-OECD country
**Population/Context**	Participants who have experience of working from home	Participants and populations who are unable to work from home, or work away from their work office in a public place (e.g., coffee shops, shared spaces)
	The sample of participants must include individuals who work from home with a desk based non-manual job	A sample of participants who only have manuals jobs (e.g., those drawn from the care setting (i.e., live in carers or nurses)
	Participants who are considered adults	Participants who are considered children, or drawn from the education setting (e.g., online students, university students)
**Outcome(s)**	The study details the impact of homeworking in relation to mental health, resilience^2^, or productivity	The study does not detail the impact of homeworking in relation to mental health, resilience, or productivity

^1^Any study methodology/design (i.e., qualitative, quantitative, or mixed) including primary research was eligible for inclusion

^2^Resilience as defined as ‘positive adaptation in response to adversity’ [9]

### Information sources

#### Electronic database searches

Search terms were created in relation to population/context, intervention, and outcome of the research question, as recommended by Cochrane’s Handbook for Systematic Reviews [11]. Terms were developed a priori from current literature and developed iteratively by the research team using preliminary searches to ensure a manageable and focused scope of investigation.

The final search was conducted on the 25^th^ of January 2022 across the following databases:

- Ovid®SP MEDLINE.® 1946 to January 18, 2022
- Ovid.®SP Embase 1974 to 2022 January 14
- Ovid.®SP APA PsycINFO 1806 to January Week 2 2022
- Web of Science™ Core Collection

The final search involved two strings of terms: firstly, those relating to homeworking, and secondly, psychological terms encompassing mental health, resilience, and productivity. Where possible, databased controlled vocabulary was used. Free text terms remained consistent across all four searches, only differing on database specific truncation and use of punctuation. Free text terms were searched within titles and abstracts on Medline, Embase and APA PsychINFO. Free text terms were searched within title, abstract, author keywords and Keywords Plus in Web of Science Core Collection. All searches were limited to 2020 – current, to only capture data related to working from home during the COVID-19 pandemic. Full search strategies for all databases, including filters and limits used can be found in Supplemental Table 1.

#### Grey literature searches

The following sources were searched on the 1^st^ of February 2022: Google Advanced Search, NHS Evidence; Gov.uk Publications; and the British Library directory of online doctoral theses (EThOS).

The following search was used for the Google Advanced Search, NHS evidence, and EthOS. For the Google Advanced Search, the results were ordered by most relevant, and the first 20 pages (totalling 200 hits) were screened. The NHS search was limited to primary research only.

1. (“work from home” OR “telework” OR “homework”)
2. (“mental health” OR “productivity” OR “resilience”)
3. 1 AND 2

The remaining searches were kept relatively simple due to small numbers of papers available shown during preliminary searches. Gov.uk Publication searches were limited to: ‘research’ or ‘statistics’ or ‘policy papers and consultations’, including the terms “homework”, “telework”, or “work from home”. Office for National Statistics searches were “homework”, “telework” or “work from home”. Full search strategies for all registers and websites, including filters and limits used can be found in Supplemental Table 2.

### Study selection

**Fig. 1:**
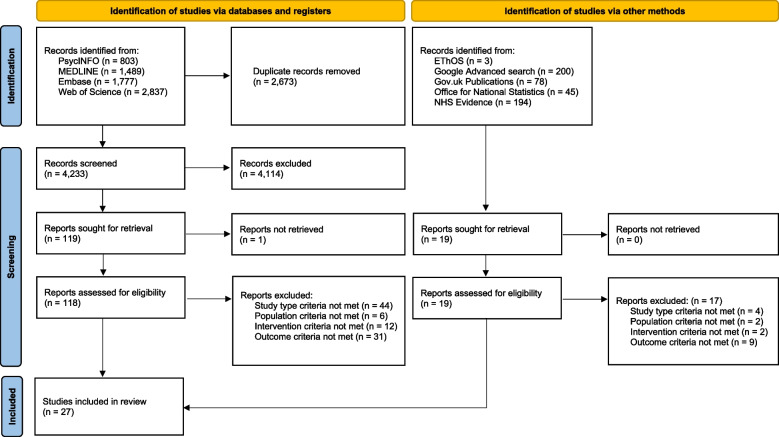
PRISMA flow diagram

Results of the literature searches were downloaded to EndNote X9 reference management software (Thomson Reuters, New York, United States (US)). Initial screening was carried out for all titles and abstracts against the inclusion and exclusion criteria by one author (CEH). Each study was categorised into one of the following groups: “include”, “exclude” or “unsure”. A 10% check of excluded papers (~ 400 records) was carried out by a second reviewer (LD), any papers marked as potentially relevant by LD were then rescreened by CEH. Both of the “include” and “unsure” categories then were subject to full text screening. To provide robustness to the review process, 10% of the papers were also full text screened by a second reviewer (LD). When there were disagreements between reviewers (i.e., on 3/12 papers), a third reviewer (SKB) was used, and the majority decision taken. Articles were then categorised into “include” or “exclude”. A PRIMSA flowchart of the screening process is presented in Fig. 1.

### Data extraction and synthesis

Data was extracted using a data extraction spreadsheet by one author (CEH). Article data and information extracted included: authors; title; type of document (e.g., publication, governmental report); publication year; publication origin; aims and hypotheses; size of sample; sample demographics and characteristics; variables of interest examined, outcome measures; key findings, limitations, and recommendations. Extraction of this data allowed for study characteristics (e.g., date of publication, country of origin, sample characteristics, outcome measures) to be reported alongside key findings, whilst considering reported study limitations and recommendations/implications suggested by the authors. A 20% check of extracted data relating to key findings was carried out by LD, no discrepancies found between reviewers. Narrative synthesis was used to collate findings from the retained papers [12]. Research findings were firstly grouped by variables examined (e.g., productivity or mental health focused), and a narrative was synthesised.

### Quality assessment

The Mixed Methods Appraisal tool [13] was used to appraise the quality of included studies based on the information provided in the papers. This tool was chosen due to its ability to appraise both qualitative and quantitative studies whilst also accounting for the differences between types of study. Many reviews have used this tool for quality assessment, for example [14–16]. Papers were checked for suitability using the following screening questions: “Are there clear research questions?”; “Do the collected data allow to address the research questions?”. Each study was then assessed using five questions relevant to the methodological approach used within the paper [13]. One author carried out the quality appraisal (CEH).

## Results

### Study selection

In total 6,906 search results were extracted from electronic databases. Post duplication screening, 4,233 papers remained for title and abstract screening. 119 papers were sought for retrieval, one paper [17] was deemed potentially relevant to the review, but after exhausting all means of accessing the full text the paper had to be excluded from the review. Following title and abstract screening, 118 full texts were screened, and 25 studies were retained as they aligned with the inclusion criteria. Two additional studies were included as a result of grey literature searches. Therefore, 27 studies were included in this review (refer to Fig. 1 for flow diagram).

### Study characteristics

#### Date of publication

No papers included in this review were published prior to 2020, as per the exclusion criteria. Only one paper was published in 2020 [18], 25 papers were published in 2021 [19–43], and one paper was published in 2022 [44].

#### Country of origin

Data extracted relating to the location of the first authors institution at the time of publication was extracted to display geographical spread of the papers retained within this review. As per the inclusion criterion, all paper origins are from OECD countries. The location of papers is relatively varied, with four papers originating from each of the USA [21, 28, 30, 43], the UK [19, 39, 40, 42] and Japan [32–34, 38]. Three papers originated from Turkey [26, 27, 37], and Italy [18, 22, 24]. Two papers originated from Columbia [23, 35]. The remaining papers originated from Canada [31], Germany [44], Luxembourg [36], the Netherlands [41], Portugal [20], Spain [25] and Sweden [29].

#### Study design

The majority of the retained papers used similar methodological approaches to collect data; 24 out of 27 of the papers used online surveys [18, 20–25, 27–43]. It is necessary to note that, three of these papers used additional qualitative elements in their surveys [39, 40, 42], and four surveys collected data at multiple time points [36, 38, 41, 44]. Of the remaining three papers, two used secondary data analysis [26, 44], and one paper [19] used semi-structed interviews to collect data.

#### Variables examined and measures

Of the 27 papers, 13 focused specifically on mental health outcomes [22, 24–26, 28, 29, 33, 34, 36, 37, 41–43], six on productivity outcomes [20, 21, 23, 27, 31, 32], and eight included both mental health and productivity outcomes [18, 19, 30, 35, 38–40, 44]. All measures used varied across studies with many being unvalidated. Table 2 shows more in-depth details about variable measures.

**Table 2:** Extracted information relating to outcome variable and measure, and quality appraisal score

Reference	Measures	Outcomes		Quality appraisal score (%)
		**Mental Health**	**Productivity**	
[21]	Depression, Anxiety and Stress Scale-21 items (DASS-21)	Depression Anxiety Stress		60
[23]	General Health Questionnaire (GHQ-12) 5-item World Health Organization Well-Being Index (WHO-5)	Psychological distress Subjective wellbeing		100
[24]	General Health Questionnaire	Psychological wellbeing		100
[25]	GHQ12	Mental wellbeing		60
[27]	Perceived Stress Scale-10 Copenhagen Burnout Inventory (CBI)	Stress Burnout		60
[28]	General Health Questionnaire, work stress questionnaire,	Health Work-related stress		60
[32]	Kessler 6	Depression Anxiety		60
[33]	Kessler 6	Psychological distress		60
[35]	“Overall, in the past week, how satisfied have you been with your life? and in the past week, to what extent have you felt the things you are doing in your life are worthwhile?” UCLA Loneliness 8 item scale PHQ9 GAD7	Wellbeing made up of: Life satisfaction / Loneliness / Depression / Anxiety		80
[36]	Depression Anxiety Stress Questionnaire-Short Form,	Depression Anxiety Stress		60
[40]	GHQ-12 five items	Psychological distress		60
[41]	“Warr’s scales (items based on asking respondents to rate the extent to which they felt (four) states in the last seven days: the states being “anxious”, “worried”, “at ease”, “relaxed”. Responses were given on a five-point scale, “never”, “occasionally”, “some of the time”, “most of the time”, and “all of the time”, and item responses were recoded such that high scores indicated better well-being. Depression–enthusiasm was measured in the same way as anxiety–contentment, with the states being “depressed”, “gloomy”, “happy” and “cheerful”). “ “Warwick–Edinburgh Mental Well-being Scale (which was adapted to fit the weekly survey, in which respondents were asked to rate during the last 7 days the extent to which they felt seven states. The states were (a) “optimistic about the future”, (b) “feeling useful”, (c) “feeling relaxed”, (d) “dealing with problems well”, (e) “thinking clearly”, (f) “close to other people”, (g) “able to make up my own mind about things”. A five-point response scale was used: “none of the time”, “rarely”, “some of the time”, “often”, and “all the time”. Thus, high scores on this measure indicated better well-being).”	Anxiety contentment Depression-enthusiasm Mental wellbeing		60
[42]	“Participants rated their overall mental well-being relative to their health status prior to WFH on a 5-point Likert-type scale, from 1 (much lower) to 5 (much higher) with 3 indicating the same as before WFH.” “To explore primary contributors to these ratings, participants indicated what type of mental health issues they were experiencing. (Eight types of mental health issues were also provided as options: anxiety or nervousness; depression, sadness, or participants rated their overall physical and mental well-being relative to their health status prior to WFH on a 5-point Likert-type scale, from 1 (much lower) to 5 (much higher) with 3 indicating the same as before WFH. feeling blue; insomnia or trouble sleeping; low motivation or slowed actions; mental stress, rumination, or worry; mood swings; social isolating or decreased interest in social engagement; and trouble concentrating, maintaining attention or focus).”	Mental wellbeing + additional information		60
[20]	“Respondents rated their productivity relative to the status before WFH using a 5-point Likert scale with 1 indicating much lower productivity, 3 indicating the same as before, and 5 indicating much higher productivity.”		Productivity	60
[22]	“Talukder et al., questionnaire: 10 items (one of which was eliminated since its outer loading registered below 0.5; e.g. ‘I meet formal performance requirements of the job’, ‘I can make constructive suggestions to the overall functioning of my work group’).”		Job performance	80
[26]	“We questioned the total duration of working from home after the pandemic started, the level of stress or comfort compared with the workplace, productivity compared with the workplace, quality of work compared with the workplace.”		Productivity	60
[30]	Measures not disclosed		Productivity	20
[31]	"Suppose your productivity at your normal workplace is 100, how do you evaluate your work productivity at home? Please answer this question considering all of your tasks—if higher, please answer with a score higher than 100."		Productivity	40
[19]	Hospital Anxiety and Depression Scale “Perceived productivity in comparison to the participants’ previous experience in presential work was assessed qualitatively on a 7-point ordinal scale ranging from the same level of productivity to increased or decreased productivity (‘slightly’, ‘moderately’ and ‘extremely’).”	Anxiety Depression	Perceived productivity	60
[29]	Shirom and Melamed’s (2006) burnout scale “Two survey items assessed productivity related to work duties. E.g., “I can finish a large number of work-related tasks daily.” Both items were measured on a 7-point Likert scale, from 1 = “Strongly disagree” to 7 = “Strongly agree.””	Burnout	Productivity	60
[17]	"Asked about factors that might improve productivity (saved travel time to go to the office, time flexibility, autonomy, reconciliation of work life with personal and family life, enhanced attention) or might decrease it (distractions in the domestic environment such as children to look after, planning di ficulties, impaired interaction with colleagues, technical failures)."	Work-related stress	Productivity	40
[43]	Burnout Bullying Inventory Work Ability Index	Burnout	Level of work ability	40
[34]	Five items from Folkman and Lazarus’s (1985) Work Stress Questionnaire " Respondents were asked to compare remote work and previous on-site-jobs and answer: My work productivity has... using better, the same, or worse as responses"	Work stress	Work productivity	60
[37]	Brief job stress questionnaire Work Limitations Questionnaire	Job stressors and stress responses	Presenteeism	60
[38]	“Two straightforward questions aimed to capture respondents’ overall experiences of how their mental health had changed since WFH”	Mental Health	Work performance	60
[39]	Kessler—6 Distress Scale Brief Instrument to Assess Workers' Productivity During a Working Day	Mental Health	Work productivity	60
[18]	Qualitative research	Qualitative research	Qualitative research	100

#### Study sample

There was substantial variation in the sample characteristics across the included papers. Sample size varied highly between papers, ranging from *n* = 32 [19] to *n* = 20,395 [34]. In relation to job role, many papers included participants from difference sectors and occupations within their study [19, 21–23, 25, 27, 28, 31–33, 37–39, 41, 43, 44], two included a representative participant group [26, 36], some targeted specific occupations or groups (e.g., Alumni from the Portuguese AESE Business School [20]; Italian professionals [24]; university staff [29, 42]; behaviour analysists [30]; administrative workers [18]) and, some did not provide information on job role but focused on home working populations [34, 35, 40]. Table 3 displays extracted data in relation to sample size and characteristics including location and job role details.

**Table 3:** Extracted information relating to sample characteristics

Reference	Participants				
	**n**	**Job**	**Characteristics**	**Location**	**Additional**
[18]	32	"The participants come from different sectors and occupations, including UK higher education, accounting and finance, sales, marketing and project management"	17 females Age range: 25–47	UK	• 3–12 years work experience • Sample was representative of the study population • *n* = 18 married
[19]	143	"sample of alumni from the Portuguese AESE Business School"	Males 56% Average age: 49.2	Portugal	• Most individuals completed bachelor, master or doctoral degrees (94%) • Study population (*N* = 143) was a homogeneous sample regarding most sociodemographic variables • Marital status (married 72%) • Presence of children (88%)
[20]	988	"Respondents primarily worked in occupations categorized as business and office (29.1%), engineering and architecture (24.6%), education and arts (22.1%), followed by respondents in healthcare and social services (9.3%), computer sciences and mathematics (8.2%), basic scientists (4.2%), and services and physical occupations (2.6%)."	558 women 317 men 113 unreported Age range: 18–80 years old	U.S (10.5% of respondents did not provide their location)	• Respondents were primarily Caucasian (60.9%)
[21]	804	"finance, insurance and banking services (7.1), entrepreneurship and management 50 (6.2), communication (10), education and research 129 (16), public administration and law enforcement agencies 43 (5.3), IT and telecommunication 130 (16.2), health and social services 104 (12.9), real estate, design and fashion sectors 25 (3.1), industry and trade 55 (6.8), commercial services 48 (6), legal and administrative services 34 (4.2), entertainment and personal services 18 (2.2), consulting services 31 (3.9)"	Males 40% Mean age: 39.2	Italy	• Education N (%): Middle school level 9 (1.1), High school license 145 (18), Bachelor degree 601 (74.8), Doctoral degree 49 (6.1)
[22]	519	"23 large, private, service-sector companies wholesale and retail trade, transport, financial services, education, health care, and real estate"	Female 71% Age 20–30 (11) 31–35 (17) 36–45 (50) 46–50 (18) 55 or older (4)	Columbia	• The questionnaire was provided to 519 teleworkers occupying mid-level positions in their organizations who had been using this modality before the pandemic at least two days a week
[23]	575	"participants belonging to an association of Italian professionals"	Females 60.52%, Males (39.48%) Median age of 40 years (IQR: 33–49)	Italy	• "Most of the participants were graduated or post-graduated (73%) • Most of the responders were employees (65.22%) with a median job seniority of 9 years 54 (9.39%) were 24 h available, while the others had ordinary or flexible shifts."
[24]	1050	"scientific and intellectual professionals, support and administrative employees, accounting and administrative employees, catering, protection and sellers, other lower-skills workers"	“The percentage of women is higher than men for […] teleworking individuals.”	Italy	• To be included participants must: have been confined in Spain, been in the labour market, and having had some paid work experience during the last 12 months
[25]	14,520 observations and 3630 individuals when we considered those who work only from home, and 12,144 observations and 3036 individuals when we considered the respondents who work from home on occasion	All data from: Understanding Society-UKHLS, a nationally representative survey of approximately 30,000 households started in 2009		UK	
[26]	194	Engineer, Analyst, Academician, Software specialist, Banker, Marketing expert, Interior architect, Executive, Intern, Consultant, Doctor	51% men	"The study was conducted in accordance with the principles of the Declaration of Helsinki."	• All the participants had no previous experience of working from home and only started working from home after the pandemic
[27]	256	"Manager/Supervisor (29%), Educator (12%), Professional (12%), Executive (6.5%), Student (3%), or Other (20%). In the “Other” category, “Administrator,” “Director,” and “Researcher” were the most frequent responses."	“The final sample [was] reflective of the general population in terms of gender identity (49% female).”	not specific	• The group was well-educated with over 40% of the sample having a graduate degree or higher
[28]	392	An electronic questionnaire was sent to junior lecturers, senior lecturers, and professors at Swedish public universities	The sample contained a higher proportion of women than the population (62% in the sample and 52% in the population)	Sweden	• The proportion of junior lecturers in the sample was similar to that in the population (45% in the sample and 44% in the population) • The proportion of senior lecturers was slightly larger (44% in the sample and 42% in the population) • The proportion of professors was smaller (11% in the sample and 14% in the population)
[29]	491	Applied behaviour analysts	Female 89% Average age: 33.45 years	USA	• Participants reported being employed for an average of 34.08 h per week • During that time, approximately 54% of participants were working remotely as a result of COVID-19 • The majority of respondents had BCBA® certification (62%) • 84%of the participants identified as non-Latino white
[17]	51	All individuals were employed as administrative wo rkers that moved to work remotely since the beginning of COVID-19	Women 56.9% Average age: 46.67 years	Italy	• Most of the participants had three or more cohabitants (56.9%) • 29.4% had children to look after • 55% of workers had a second level degree
[31]	5105	Roles consisted of: agriculture, construction, IT, transport, wholesale and retail, finance and insurance, real estate, accommodation and restaurants		Japan	
[43]	519	Participants were from: 12.7% other services; 10.4% other industries; 9.4% IT, computers, and mathematics; 8.3% social work and education; 8.1% office, business, and administration; 7.1% banking, insurance, and real estate; 6.4% health, medicine, nursing, and sports; 5.6% trade, distribution, sales; and all other industries < 5%	46.4% female Average age was 45.37	Germany	• Individual data from a large Germany-wide study was used to investigate the research questions • University and technical college degrees were most frequently reported as the highest professional education (39.3%) • The average monthly net income (i.e., the sum of wages, salary, income, in each case after deduction of taxes etc.) was 2000–3000
[32]	1896	General workers from companies that had business contact with BackTech Inc. The survey was administered to 5,000 subjects	76.8% male Average age: 44.52	Japan	• Of the 1,896 people who responded to our survey on the status of telework, 1,597 (84.2%) had switched from office to telework, and 213 (11.2%) teleworked unrelated to COVID-19 • The remaining demographic indicators were statistically different, such as residence, marital status, cohabitant presence, managerial position, and employment status
[33]	20,395	Job type: Primarily desk work 2042 (80.5%)	55.9% male Average age: 49.5	Japan	• 12% telecommuted more than 4 days per week, 1,391 (7%) telecommuted more than 2 days per week, 1,343 (7%) telecommuted less than 1 day per week, and 15,124 (74%) hardly ever telecommuted • Married (50.7%)
[34]	1285	The only inclusion criteria were that the respondent was teleworking at the time	65.9% female Average age was 29.1	Columbia Most of the respondents were from Colombia (54.8%), 39.7% were from Ecuador, and 5.5% were from other countries of the region	Of those, 68.6% were married and 49.3% lived with children. About a third of the sample had a college degree (33.8%) and 60.8% had a graduate education. Most of them worked in education (44.4%) and the second-best represented sector was service (18%). Finally, 89.5% of the respondents had been in their current job more than a year
[35]	9700	Not specific	52% male Average age was 43.5	France, Germany, Italy, Spain or Sweden	• Data from COME-HERE (COVID-19, MEntal HEalth, REsilience and Selfregulation) panel survey run by the University of Luxembourg • This survey is conducted by Qualtrics using representative samples (by age, gender and region) from France, Germany, Italy, Spain and Sweden
[36]	459	100 (21.8%) were software developers, 79 (17.2%) were pharmaceutical industry employees, 74 (16.1%) were sales and marketing employees, 67 (14.6%) were bank employees, 29 (6.3%) were public officers, 29 (6.3%) were engineer, 24 (5.2%) were textile workers, 11 (2.4%) were airlines workers, 11 (2.4%) were insurance workers, 9 (2.0%) were teachers, 9 (2.0%) were food workers, 8 (1.7%) were logistics workers, 6 (1.3%) were human resources workers, 3 (0.7%) were tourism professionals	Out of 459 respondents, 254 (55.3%) were male and 205 (44.7%) were female. The age of the participants ranged from 24 to 60 years (M = 35.64, SD = 6.84) and mean age was 35.64 ± 6.84	As far as education level is concerned,	• Primary inclusion criteria for the participants were no remote working experience prior to the COVID-19 pandemic, WFH for at least six months after the COVID-19 pandemic declaration, WFH at the time of the questionnaire • 318 (69.3%) participants were graduated from university, while 141 (30.7%) were postgraduate degree holders • 28 (49.7%) of the participants had a child
[37]	3123	40 companies information technology, finance, broadcasting, music, consulting, public office, chemical industry, healthcare, fashion, printing, movie, trading, restaurant, travel agency, patent agency, and temp agency	1,773 males Average age: 37.3	Japan	• 1,440 participants (46.1%) had not engaged in remote work in 2020. Among the other participants, 713 people (22.8%) had engaged in 1 or 2 days a week of remote work, 728 people (23.3%) had engaged in 3 or 4 days a week of remote work, and 242 (7.7%) people had engaged in 5 days a week of remote work
[39]	184	Recruited entirely online. No job category data reported	Men (*n* = 40) and women (*n* = 143)	UK	• Households with (*n* = 46) and without (*n* = 136) children under the age of 18 years
[40]	1164	"Most of our respondents worked in the public administration sector (45.3%); other sectors included support services (10.2%), professional services (9.1%), information and communications (8.1%), education (5.1%) and manufacturing (4.6%)"	Female 76.6% Average age: 46.45	Finland	• Most of the respondents were part of two-person households (42.4%); others were part of one-person (19.8%), three-person (16.2%) or four-person (15.1%) households • 34.3% of the respondents had at least one child (< 18 years) living at home
[41]	831 first phase **492 s phase**	"Administered to employees at two universities in England"	Female 74% /** 77%** Under 30: 10% /** 11%** Over 50: 30% /** 33%**	UK	• Graduate: 86% /** 86%** • White: 89% /** 92%** • Married/living with a Partner: 74% / **73%** • Childcare responsibilities: 25% /** 23%**
[42]	988	"Respondents worked across a variety of occupations including those in business and office (29.1%), engineering and architecture (24.6%), education and arts (22.1%), healthcare and social services (9.3%), computer sciences and mathematics (8.2%), basic science (4.2%), and service and physical occupations (2.6%)."	Female 56.6% Male 32.1% Prefer not to say 11.4% Average age: 40.9	USA "individuals working in California (47.3%), with additional responses received from 39 other states in the U.S. (35.8%) and countries outside of the U.S. (6.4%), and the remaining 10.5% of respondents preferring not to answer."	• Race or ethnicity of the respondents included Caucasian (60.9%), Asian (24.6%), Hispanic or Latino (9.3%), African American (2.8%), and mixed race or another ethnicity (2.4%) • One-third of respondents reported having either a doctorate (34.1%) or graduate/ professional degree (37.2%), while the remaining respondents had either a 4-year degree (22.1%) or a 2-year degree or less (6.5%)
[38]	3140	The business/industry sector of the 2,768 who responded are as follows: telecoms (24.1 per cent), local government (17.9 per cent), financial services (16.6 per cent), civil service (14.9 per cent), education (4.2 per cent), voluntary/third sector (4.1 per cent), travel/transport (3.5 per cent), NHS (2.1 per cent) and others (5.1 per cent). Of the 2,985 who responded, 30.5 per cent were contact centre workers	66.4% female, 31.3% male, 0.4% non-binary 1.4% preferred not to state their gender Age: < 25: 3.1% 26–35: 15.4% 36–45: 20.7% 46–55: 33.8% 56–65: 25.5% 65 + : 1.4%	UK	• full-term permanent (76.3%), part-time permanent (21.4%), full-time temporary (0.7%), part-time temporary (0.1%), fixed terms contracts (1.2%), zero hours contract (0.2%) • The grade of the 3014 respondents were as follows: senior management (3.4%), middle management (14.3%), team leader/supervisor (10.4%) and non-managerial/non- supervisory (71.9%)
[30]	2758	The focus is on employees aged 15 to 64 who are new teleworkers, i.e., who usually worked outside the home prior to the COVID-19 pandemic but worked most of their hours at home during the week of February 14 to 20, 2021 Goods-producing industries; Trade, transportation and warehousing; Finance and insurance; Professional, scientific and technical services; Education, law and social, community and government services; Health care and social assistance; Public administration; Other	Not reported	Canada	

#### Quality appraisal

Overall quality of papers varied across the 27 that were retained, with an average score of 62%. The MMAT quality scores as a percentage can be found in Table 2. The included papers within this systematic review varied in quality. Many were cross-sectional, quantitative in methodology, and recruited participants using snowball or opportunistic sampling. This resulted in some unclear sample characteristics (e.g., not knowing where a percentage of participants were from), and uncertainty as to how often the sample were working from home. Only three of the retained papers within this review used qualitative research elements, and there was no common method for measuring mental health, or productivity across homeworking research.

#### Synthesis

To allow comparisons across and between research, findings relating to mental health and productivity will be separated and reported on separately in the following section.

#### Mental health

This following section details outcomes relating to mental health and synthesises the following outcomes from 21 papers: ‘depression’ [20, 22, 33, 37, 42]; ‘anxiety’ [20, 22, 33, 37, 42]; ‘stress’ (including work stress) [18, 22, 28, 29, 35, 37, 38]; ‘psychological distress’ [24, 34, 41]; wellbeing [36] (including ‘subjective wellbeing’ [24], ‘psychological wellbeing’ [25]; ‘mental wellbeing’ [26, 42, 43]); ‘health’ [29]; ‘burnout’ [28, 30, 44]; and general ‘mental health’ [39, 40]. Table 2 provides additional information on how these outcomes are measured, and it is necessary to note that there are overlap in how outcomes are described (i.e., ‘mental wellbeing’, ‘psychological wellbeing’, ‘health’, and ‘psychological distress’ were all measured using the same questionnaire).

The findings in relation to mental health varied across the retained papers. Many of the papers reported a negative relationship between homeworking and mental health and wellbeing [19, 24–26, 29, 30, 33, 36–41, 43, 44]. For example, one paper established that the transition to homeworking during the pandemic increased psychological strain due to increased work intensification, poor adaptation to new ways of working, and online presenteeism [19]. Another paper reported that out of those who continued to work during the COVID-19 pandemic (i.e., not furloughed, or unemployed), teleworkers experienced less self-perceived wellbeing than those who continued working at their pre-COVID-19 workplace [25].

Some of the retained papers concluded a mixed findings in relation to home working and mental health. For example, despite a main finding that working from home during the COVID-19 pandemic results in lower levels of well-being, Schifano et al., also concluded that when the sample only includes those who switched to homeworking from office working, there is a small fall in anxiety levels when moving to working from home [36]. Additionally, Taylor et al., reports that around 40 per cent believe that their mental health had worsened either a lot or a little since working from home, compared to around 30 per cent that believed their mental health had improved [39]. Similarly, Moretti et al., reports that around 40 per cent of participants declared a reduced stress level since they have worked remotely, around 30 per cent reported an unchanged level, and one-third of participants experienced increased stress [18].

Homeworking was found to have no association with burnout by one retained paper [30]. Shimura et al., provides evidence that remote work does decrease psychological and physical stress responses when controlling for confounding factors such as job stressors, social support, and sleep status [38]. Working from home was also considered to be better for wellbeing in comparison to being furloughed or unemployed [25, 36].

### Factors affecting mental health when homeworking

#### Demographics

When considering age, findings were mixed. One paper reported being older [36] resulted in poorer mental health outcomes. Additionally, another paper focused on stress and burnout specifically reported that being a young male [25–34], an older male (55 +) or a middle aged or older woman (45 +) resulted in increased stress, and being a middle-aged man [35–54] increased burnout [28].

Being female was reported to result in increases of depression, anxiety, and stress [37]. Females were also reported to experience two or more new physical or mental health issues were provided in comparison to male workers [43]. In this study, nine types of physical issues were assessed, these included, but are not limited to, musculoskeletal discomfort or injury, headaches or migraines, cardiovascular issues. Eight types of mental health issues were assessed, these included, but are not limited to, anxiety or nervousness, mental stress, rumination or worry, depression, sadness, or feeling blue [43].

#### Occupation

Those considered better-educated were reported to have worsened mental health outcomes [36]. Those working in the field of “education and research” judged their telework experience to be much worse than participants working in other fields (e.g., ‘IT and telecommunication’, ‘Public administration and law enforcement agencies’, ‘Health and social services’ and ‘Legal and administrative services’) and were less willing to replicate the telework experience, there were also higher levels of stress and anxiety apparent [22].

#### Living arrangements

Living and working in a home which is considered crowded or confined resulted in poorer mental health [33, 36]. Having a larger house and living with a partner, or with one or two housemates, was also found to be protective of mental health [22].

Results are mixed in relation to working in a household that includes children. On one hand, having young children in the home was considered to have a negative link to wellbeing, supposedly related to increased demands [36]. Whereas other research reported having infants (less than two years old) or toddlers (two to five years of age) at home as protective of wellbeing but were also associated with more mental health issues [43]. These conflicting findings were reasoned to be due to working parents being able to spend more time at home with their children, resulting in better mental wellbeing. However, due to work-life strain caused by increased demands and lack of support (i.e., from babysitters) during working hours there is an increase in new physical and mental issues apparent [43].

#### Isolation or loneliness

Spending more time remote working was considered to increase perceptions of isolation, and isolation and psychological distress were reported to mutually affect each other over time [41]. Additionally, having frequent contacts with work colleagues was considered protective factors of mental health [22].

#### Homeworking preference

Workers who preferred to work from home experienced less psychological distress with increasing telecommuting frequency, while those who preferred not to telecommute experienced more psychological distress with increasing telecommuting frequency [34].

#### Length of time homeworking

The association between working from home and mental health and wellbeing was found to differ depending on frequency and length of time home working [26, 29, 33, 44].

One paper found working from home for a short duration was considered no different on mental well-being in comparison to those always working at the employer’s premises [26]. Niu et al., found that there was initially no difference in the mental health between workers who continued working in the office and those who switched to telework, but participants who teleworked for a longer period showed more severe anxiety and depression in comparison to those who teleworked for a short period. [33]. Similarly, those working from home for a high percentage of their weekly hours reported more negative psychological symptoms than employees who work from home for less hours [44], and higher ratings of stress were also reported in those working from home several times per week in comparison to those who worked from home less than once per month [29].

### Productivity

This following section details outcomes relating to productivity and synthesises the following outcomes from 14 papers: ‘productivity’[18, 21, 27, 30–32, 35, 40], ‘performance’ [23, 39], ‘percieved productivity’ [20], ‘level of work ability’ [44], ‘presenteeism’ [38]. Table 2 provides additional information on how these outcomes are measured.

The findings in relation to productivity varied across the retained papers. Some of the retained papers concluded a negative relationship between home working and productivity [19, 30, 32, 40]. For example, Adisa (2021) found that the transition to home working from office-based work caused increased work intensification, online presenteeism and employment insecurity – which resulted in psychological strain and poor levels of work engagement [19]. Similarly, increased work intensity (e.g., receiving more information from teams and engaging in more planning activities) due to working from home also resulted in decreased worker productivity [30]. Morikawa et al., concludes that productivity whilst working from home was about 60–70% of the productivity at business premises, and was especially low for employees and firms that started homeworking after the onset of the COVID pandemic [32]. A UK-wide survey of office workers (including telecom, local government, financial services and civil service staff) who were working from home during the COVID-19 pandemic reported that since the onset of homeworking, 30% reported of workers that it is now more difficult to meet targets, and they had concerns of underperforming [39].

Some studies concluded that working from home was in fact no different in comparison to office working in terms of productivity [23]. This was reported for those who worked at home pre-COVID-19 and tended to practice working from home frequently [32]. Additionally, other research concluded that 90% of new teleworkers reported being at least as productive (i.e., accomplishing at least as much work per hour at home) as they were previously in their usual place of work [31].

Moretti et al., reported that working at home resulted in productivity decreasing in 39.2% and an increasing in 29.4% of participants [18]. However, Guler et al., established that participants who worked from home were more relaxed, more efficient, and they produced better quality work [27]. Despite reported increased or no change to levels of productivity, some research studies did find that those working from home were reporting longer working hours [21, 27].

### Factors affecting productivity when homeworking

#### Demographics

Two papers reported that males were less productive than females when working from home [20, 21]. Those who are older and have higher levels of income are also more likely to be productive when homeworking [21], as were those who are unmarried with no children [31]. Those who are highly educated, high wage employees, long distance commuters, tended to exhibit a relatively small reduction in productivity [32]. Having an appropiate workspace was also associated with higher levels of productivity [21].

#### Occupation

In terms of occupation, “scientists” were most likely to have the highest level of productivity, in comparison to “engineering and architecture,” “computer sciences and mathematics” and “healthcare and social services.” [21]. Other research also supported that those who work in in information and communications industry only displayed a relatively small reduction in productivity [32]. Higher levels of productivity in were also apparent in public administration (41%) as well as in health care and social assistance (45%). In contrast, the corresponding percentage was lower in goods-producing industries (31%) and educational services (25%) [31].

### Mental health and productivity

A few of the retained studies looked at the interaction between mental health and productivity whilst homeworking [21, 27, 35]. In a sample of staff that had been working from home for more than 6 months, it was reported that they were less stressed, more efficient, and had better quality of work during working from home period according to self-report data [27]. Other research reported that having an appropiate workspace, and better mental health was also associated with higher levels of productivity [21]. Stress was also found to lessen the positive association between working remotely on productivity and engagement [35].

## Discussion

This systematic literature review sought to 1) explore the association between working from home and both, mental health, and productivity, and 2) establish potential risk factors. Literature searches encompassed both peer previewed published literature and grey literature, 27 papers were retained post screening and included within this review. The results established that relationship between homeworking and both, mental health and productivity varies considerably, suggesting a complex association with many mediating and moderating factors.

Prior to the COVID-19 pandemic and the introduction of enforced and prolonged homeworking, working from home was often considered advantageous. Research often concluded that homeworking had multiple advantages [4, 45–47]. There were also potential concerns reported with homeworking [45, 48], for example in relation constant connectivity to the workplace [5], but these were not considered to outweigh the benefits [48]. This review revealed conflicting findings, with the majority of the research suggesting a negative or mixed link to mental health, which is supported by current literature [6].

This suggests that homeworking as a choice is considered largely beneficial (i.e., as shown by research prior to the pandemic), but when homeworking is instead mandatory there is potential that it may have a more negative association for certain individuals and occupations over others.

The relationship between working from home and productivity was also mixed, in that some papers found that home workers could be more productive, whereas others found the opposite. However, most studies reviewed show that homeworking for both new starters (e.g., has only worked from home) and those transitioning to homeworking for the first time, were particularly likely to report low levels of productivity along with concerns about meeting targets. There was also consistency amongst reviewed papers that homeworkers who reported better mental health (e.g., were less stressed) were more productive which is consistent with previous research showing an inverse relationship between stress levels and productivity [49, 50]. Taken together, findings from the current review suggest that prolonged homeworking can negatively affect mental health, and in turn, lower levels of mental health can negatively affect productivity. Therefore, there should be a focus on maintaining and mitigating workers mental health when they are asked to work from home for a prolonged period.

Feelings of isolation or loneliness in homeworkers were also considered to have a consistent link to poorer mental health. This finding is well supported as the negative association isolation and loneliness have on mental health is widely reported across research (e.g., [51, 52], and as demonstrated in an overview of systematic reviews [53]). The ability to create a shared sense of social identity with colleagues, which is protective of workplace stress [54] and burnout [55], may be hindered by homeworking [56] which can result in feelings of isolation or loneliness. This finding suggests that opportunities for social integration should be promoted by managers and team leaders. For example, through team meetings, in person events, or where possible, office working days.

As the findings relating to both mental health and productivity were varied, examination of factors which have potential to affect this relationship were explored. Personal and practical factors such as, being female, older in age, living and working in a crowded or confined home, or having young children at home were consistently associated with worsened mental health. Literature also concludes, being female, older in age, a highly educated high wage earner, being unmarried with no children, or someone with an active advantage towards homeworking (e.g., long distance commuters), and an appropiate workspace were associated with higher levels of productivity. These findings highlight the importance of considering practical factors that could be targeted by potential interventions (e.g., exploring how to manage work and having children at home, having an appropriately sized workspace, and managing overcrowded housing situations) as well as tailoring interventions to suit the target demographic (e.g., by considering gender, age, and occupation).

### Limitations

Limitations for the current review these can be split into retained paper limitations and review process limitations. In terms of retained paper limitations, quality screening established that the retained papers varied in quality. Many were cross-sectional (only four studies within the current review collected data from multiple time points), quantitative in methodology, and recruited participants using snowball or opportunistic sampling. This resulted in some unclear sample characteristics (e.g., not knowing where a percentage of participants were from), and uncertainty as to how often the sample were working from home. These elements limit the generalisability of the findings, and this should be considered when conclusions are drawn from this data.

For this review specifically there are a number of limitations to consider. Firstly, limiting the search to English only may have resulted in the exclusion of potentially relevant papers. Secondly, this review did not seek to collate findings from studies which only directly compared those who had to work from home during the pandemic vs. those who could not, or did not, work from home, which could have potentially provided clearer results. However, where papers provided comparisons (e.g., [25, 36]) they were extracted and presented in the results. Thirdly, current literature has established that working throughout the pandemic can be negatively related to mental health [57–59], which makes it difficult to disentangle the impact of working from home specifically. However, in the current review, three papers indicated that homeworking has potential to be negatively linked to mental health when carried out, or continued, for a long period of time (in comparison to hybrid working or working from home for a short period). This could possibly be due to the previously reported benefits of homeworking (e.g., flexibility, eradicating commuting time, and work life balance) no longer feeling advantageous when constantly working from home. This is an area that requires more research and is discussed in more detail in the following section.

### Implications and future research

The current review found that working from home is neither positively or negative related to mental health or productivity, suggesting that a one size fits all approach to tackling the mitigation and management of workers mental health and productivity whilst they work from home is not suitable nor fit for purpose. However, there are indications that those who start homeworking for the first time during a pandemic are at risk of poor productivity, as are those who experience poor mental health. This suggests that employers should aim to help those who are new to home working, for example through training or mentoring programs. Additionally, those at risk of having poor mental health should be more closely monitored and provided with early support to ensure productivity.

The varied nature of the findings also calls for more in-depth research into why homeworking has such wide-ranging effect on individuals, and what factors have potential to mitigate and moderate this relationship. Due to the wide-ranging findings, it may be sensible to focus on specific occupational contexts and qualitatively explore barriers and facilitators to working from home to provide in depth rich data. Such work is currently underway as a PhD project focused on response organisations that worked from home during the COVID-19 pandemic conducted by the first author of the current review.

Considering the impact of working from home for different durations is also important, as the current review establishes that three papers indicated that homeworking has potential to be negatively associated with mental health when carried out, or continued, for a long period of time. Further empirical research is needed to provide more detail into, this finding along with examination into the factors that could impact this relationship (e.g., isolation, pre-existing mental health concerns). Resilience factors and characteristics associated with growth and flourishing whilst working from home should also be the subject of future research.

Methodologically, future research should seek to employ qualitative or mixed method designs to collect more in-depth and complete data in relation to the psychological effect of homeworking. Additionally, there should be a focus on using similar research measures when adding to the homeworking evidence base, as this would allow for research finding to be accurately compared. Similar suggestions were reported in a recent rapid review [60].

## Supplementary Information

**Additional file 1:** **Supplemental Table 1.** Search Strategy. **Supplemental Information Table 2.** Grey literature Searches.

## Data Availability

The datasets used and/or analysed during the current study are available from the corresponding author on reasonable request.

## Abbreviation

OECD: Organisation for Economic Co-operation and Development

## References

[1] Office for National Statistics. Coronavirus and homeworking in the UK: April 2020. [Internet] Office for National Statistics; 2020. Available from: https://www.ons.gov.uk/employmentandlabourmarket/peopleinwork/employmentandemployeetypes/bulletins/coronavirusandhomeworkingintheuk/april2020.

[2] Office for National Statistics. Homeworking and spending during the coronavirus (COVID-19) pandemic, Great Britain: April 2020 to January 2022. [Internet] Office for National Statistics; 2022. Available from: https://www.ons.gov.uk/releases/homeworkingandspendingduringthecoronaviruscovid19pandemicukapril2020tojanuary2022.

[3] Felstead A, Reuschke D (2020). Homeworking in the UK: before and during the 2020 lockdown.

[4] Kelliher C, Anderson D (2010). Doing more with less? Flexible working practices and the intensification of work. Hum Relat.

[5] Matusik SF, Mickel AE (2011). Embracing or embattled by converged mobile devices? Users’ experiences with a contemporary connectivity technology. Hum Relat.

[6] Oakman J, Kinsman N, Stuckey R, Graham M, Weale VJBPH. A rapid review of mental and physical health effects of working at home: how do we optimise health? BMC Public Health. 2020;20(1):1–13. 10.1186/s12889-020-09875-z PMC7703513 33256652

[7] Lunde L-K, Fløvik L, Christensen JO, Johannessen HA, Finne LB, Jørgensen IL (2022). The relationship between telework from home and employee health: a systematic review. BMC Public Health.

[8] Elbaz S, Richards JB, Provost Savard Y. Teleworking and work–life balance during the COVID-19 pandemic: A scoping review. Canadian Psychology / Psychologie Canadienne. 2022.

[9] Luthar SS, Cicchetti D (2000). The construct of resilience: Implications for interventions and social policies. Dev Psychopathol.

[10] Moher D, Shamseer L, Clarke M, Ghersi D, Liberati A, Petticrew M (2015). Preferred reporting items for systematic review and meta-analysis protocols (PRISMA-P) 2015 statement. Syst Rev.

[11] Higgins JP, Thomas J, Chandler J, Cumpston M, Li T, Page MJ, Welch VA, editors. Cochrane handbook for systematic reviews of interventions. John Wiley & Sons; 2019. 10.1002/14651858.ED000142 PMC10284251 31643080

[12] Popay J, Roberts H, Sowden A, Petticrew M, Arai L, Rodgers M, Britten N, Roen K, Duffy S. Guidance on the conduct of narrative synthesis in systematic reviews. A product from the ESRC methods programme Version. 2006;1(1):b92.

[13] Hong QN, Fàbregues S, Bartlett G, Boardman F, Cargo M, Dagenais P, Gagnon MP, Griffiths F, Nicolau B, O’Cathain A, Rousseau MC. The Mixed Methods Appraisal Tool (MMAT) version 2018 for information professionals and researchers. Education for information. 2018;34(4):285-91.

[14] De Kock JH, Latham HA, Leslie SJ, Grindle M, Munoz S-A, Ellis L (2021). A rapid review of the impact of COVID-19 on the mental health of healthcare workers: implications for supporting psychological well-being. BMC Public Health.

[15] de Pablo GS, Vaquerizo-Serrano J, Catalan A, Arango C, Moreno C, Ferre F (2020). Impact of coronavirus syndromes on physical and mental health of health care workers: Systematic review and meta-analysis. J Affect Disord.

[16] Chen J, Farah N, Dong RK, Chen RZ, Xu W, Yin J (2021). Mental health during the COVID-19 crisis in Africa: a systematic review and meta-analysis. Int J Environ Res Public Health.

[17] Yang E, Kim Y, Hong S. Does working from home work? Experience of working from home and the value of hybrid workplace post-COVID-19. J Corp Real Estate. 2023;25(1):50–76.

[18] Moretti A, Menna F, Aulicino M, Paoletta M, Liguori S, Iolascon G (2020). Characterization of Home Working Population during COVID-19 Emergency: A Cross-Sectional Analysis. Int J Environ Res Public Health.

[19] Adisa TA, Ogbonnaya C, Adekoya OD. Remote working and employee engagement: a qualitative study of British workers during the pandemic. Inf Technol People. 2021 (ahead-of-print).

[20] Afonso P, Fonseca M, Teodoro T (2021). Evaluation of anxiety, depression and sleep quality in full-time teleworkers. J Public Health.

[21] Awada M, Lucas G, Becerik-Gerber B, Roll S (2021). Working from home during the COVID-19 pandemic: Impact on office worker productivity and work experience. Work.

[22] Bertino V, Nistico V, D'Agostino A, Gambini O, Demartini B (2021). Telework during COVID-19 outbreak: Impact on mental health among Italian workers. Eur Psychiatry.

[23] Campo AM, Avolio B, Carlier SI. The relationship between telework, job performance, work–life balance and family supportive supervisor behaviours in the context of COVID-19. Glob Bus Rev. 2021:09721509211049918.

[24] De Sio S, Cedrone F, Nieto HA, Lapteva E, Perri R, Greco E (2021). Telework and its effects on mental health during the COVID-19 lockdown. Eur Rev Med Pharmacol Sci.

[25] Escudero-Castillo I, Mato-Diaz FJ, Rodriguez-Alvarez A (2021). Furloughs, Teleworking and Other Work Situations during the COVID-19 Lockdown: Impact on Mental Well-Being. Int J Environ Res Public Health.

[26] Giovanis E, Ozdamar O (2021). Implications of COVID-19: The Effect of Working From Home on Financial and Mental Well-Being in the UK. Int J Health Policy Manag.

[27] Guler MA, Guler K, Guneser Gulec M, Ozdoglar E (2021). Working From Home During a Pandemic: Investigation of the Impact of COVID-19 on Employee Health and Productivity. J Occup Environ Med.

[28] Hayes SW, Priestley JL, Moore BA, Ray HE (2021). Perceived Stress, Work-Related Burnout, and Working From Home Before and During COVID-19: An Examination of Workers in the United States. Sage Open.

[29] Heiden M, Widar L, Wiitavaara B, Boman E (2021). Telework in academia: associations with health and well-being among staff. High Educ.

[30] Jimenez-Gomez C, Sawhney G, Albert KM (2021). Impact of COVID-19 on the applied behavior analysis workforce: Comparison across remote and nonremote workers. Behav Anal Pract.

[31] Mehdi T, Morissette R (2021). Working from home: Productivity and preferences.

[32] Morikawa M (2022). Work-from-home productivity during the COVID-19 pandemic: Evidence from Japan. Econ Inq.

[33] Niu Q, Nagata T, Fukutani N, Tezuka M, Shimoura K, Nagai-Tanima M (2021). Health effects of immediate telework introduction during the COVID-19 era in Japan: A cross-sectional study. PLoS ONE [Electronic Resource].

[34] Otsuka S, Ishimaru T, Nagata M, Tateishi S, Eguchi H, Tsuji M (2021). A Cross-Sectional Study of the Mismatch Between Telecommuting Preference and Frequency Associated With Psychological Distress Among Japanese Workers in the COVID-19 Pandemic. J Occup Environ Med.

[35] Sandoval-Reyes J, Idrovo-Carlier S, Duque-Oliva EJ (2021). Remote Work, Work Stress, and Work-Life during Pandemic Times: A Latin America Situation. Int J Environ Res Public Health.

[36] Schifano S, Clark AE, Greiff S, Vogele C, D'Ambrosio C (2021). Well-being and working from home during COVID-19. Information Technology & People.

[37] Senturk E, Sagaltici E, Genis B, Gunday TO (2021). Predictors of depression, anxiety and stress among remote workers during the COVID-19 pandemic. Work.

[38] Shimura A, Yokoi K, Ishibashi Y, Akatsuka Y, Inoue T (2021). Remote Work Decreases Psychological and Physical Stress Responses, but Full-Remote Work Increases Presenteeism. Front Psychol.

[39] Taylor P, Scholarios D, Howcroft D (2021). Covid-19 and Working from Home Survey: Preliminary Findings.

[40] Tronco Hernandez YA, Parente F, Faghy MA, Roscoe CMP, Maratos FA (2021). Influence of the COVID-19 Lockdown on the Physical and Psychosocial Well-being and Work Productivity of Remote Workers: Cross-sectional Correlational Study. JMIRx Med.

[41] Van Zoonen W, Sivunen AE. The impact of remote work and mediated communication frequency on isolation and psychological distress. Eur J Work Organ sychol. 2022;31(4):610–21.

[42] Wood SJ, Michaelides G, Inceoglu I, Hurren ET, Daniels K, Niven K (2021). Homeworking, Well-Being and the COVID-19 Pandemic: A Diary Study. Int J Environ Res Public Health.

[43] Xiao Y, Becerik-Gerber B, Lucas G, Roll SC (2021). Impacts of Working From Home During COVID-19 Pandemic on Physical and Mental Well-Being of Office Workstation Users. J Occup Environ Med.

[44] Niebuhr F, Borle P, Borner-Zobel F, Voelter-Mahlknecht S (2022). Healthy and Happy Working from Home? Effects of Working from Home on Employee Health and Job Satisfaction. Int J Environ Res Public Health.

[45] Ammons SK, Markham WTJSS (2004). Working at home: Experiences of skilled white collar workers. Socio Spectr.

[46] Fonner KL, Roloff ME (2010). Why teleworkers are more satisfied with their jobs than are office-based workers: When less contact is beneficial. J Appl Commun Res.

[47] Dockery AM, Bawa S (2014). Is working from home good work or bad work? Evidence from Australian employees. AJLE.

[48] Tavares AI (2017). Telework and health effects review. J Int J Healthcare.

[49] Adaramola S (2012). Job stress and productivity increase. Work.

[50] Seva RR, Tejero LMS, Fadrilan-Camacho VFF (2021). Barriers and facilitators of productivity while working from home during pandemic. J Occup Health.

[51] Pancani L, Marinucci M, Aureli N, Riva P (2021). Forced social isolation and mental health: a study on 1,006 Italians under COVID-19 lockdown. Front Psychol.

[52] Ramírez-Ortiz J, Castro-Quintero D, Lerma-Córdoba C, Yela-Ceballos F, Escobar-Córdoba F. Mental health consequences of the COVID-19 pandemic associated with social isolation. Columb J Anesthesiol. 2020;48(4).

[53] Leigh-Hunt N, Bagguley D, Bash K, Turner V, Turnbull S, Valtorta N (2017). An overview of systematic reviews on the public health consequences of social isolation and loneliness. Public Health.

[54] Haslam SA, Van Dick R. A social identity approach to workplace stress. Soc Psychol Organizations. 2011:325–52.

[55] Haslam SA, Jetten J, Waghorn C (2009). Social identification, stress and citizenship in teams: a five-phase longitudinal study. IJSM.

[56] Thatcher SM, Zhu X (2006). Changing identities in a changing workplace: Identification, identity enactment, self-verification, and telecommuting. Acad Manag Rev.

[57] Greenberg N, Weston D, Hall C, Caulfield T, Williamson V, Fong K (2021). Mental health of staff working in intensive care during Covid-19. Occup Med (Oxford).

[58] Hall CE, Milward J, Spoiala C, Bhogal JK, Weston D, Potts HW (2022). The mental health of staff working on Intensive Care Units over the COVID-19 winter surge of 2020 in England: a cross sectional survey. Br J Anaesth.

[59] Palma-Vasquez C, Carrasco D, Hernando-Rodriguez JC (2021). Mental health of teachers who have teleworked due to COVID-19. Eur J Investig Health, Psychol Educ Inform Tech.

[60] Oakman J, Kinsman N, Stuckey R, Graham M, Weale V (2020). A rapid review of mental and physical health effects of working at home: how do we optimise health?. BMC Public Health.

