# Supplementary material for: The relationship between homeworking during COVID-19 and both, mental health, and productivity: a systematic review
Source: BMC Psychol. 2023 Jun 27;11:188. doi: 10.1186/s40359-023-01221-3 (PMC10294311; doi:10.1186/s40359-023-01221-3)
Supplement: Supplementary file 1 — Additional file 1: Supplemental Table 1. Search Strategy. Supplemental Information Table 2. Grey literature Searches. [file 40359_2023_1221_MOESM1_ESM.docx]

**Supplemental Table 1: Search Strategy**

| **Concept** | **Combinations** | **Notes** | **Database: Ovid PsychINFO 1806 to January Week 3 2022** | | |
| --- | --- | --- | --- | --- | --- |
|  |  |  | **Search Number** | **Terms** | **Results** |
| **(1) Population / Context (Homeworkers)** | **Combine with OR** | Controlled vocabulary | 1 | exp Telecommuting/ | 500 |
|  |  | Free text (ab,ti = title and abstract) | 2 | (Work* adj3 home).ab,ti. | 5616 |
|  |  |  | 3 | WFH.ab,ti. | 13 |
|  |  |  | 4 | Flexible work*.ab,ti. | 968 |
|  |  |  | 5 | Virtual work*.ab,ti. | 298 |
|  |  |  | 6 | Mobile work*.ab,ti. | 128 |
|  |  |  | 7 | Home based work*.ab,ti. | 65 |
|  |  |  | 8 | Remote employe*.ab,ti. | 28 |
|  |  |  | 9 | E-work*.ab,ti. | 95 |
|  |  |  | 10 | Home work*.ab,ti. | 543 |
|  |  |  | 11 | Homework*.ab,ti. | 5048 |
|  |  |  | 12 | Remote work*.ab,ti. | 234 |
|  |  |  | 13 | Telecommut*.ab,ti. | 296 |
|  |  |  | 14 | Telework*.ab,ti. | 366 |
|  |  |  |  | **AND** |  |
| **(2) Outcomes (Mental health, resilience or productivity)** | **Combine with OR** | Controlled vocabulary | 15 | exp Distress/ | 25465 |
|  |  |  | 16 | exp Mental Health/ | 77315 |
|  |  |  | 17 | exp Well Being/ | 51783 |
|  |  |  | 18 | exp Stress/ | 127740 |
|  |  |  | 19 | exp Mental Disorders/ | 918957 |
|  |  |  | 20 | exp Resilience (psychological)/ | 17375 |
|  |  |  | 21 | exp Employee Productivity/ | 3561 |
|  |  |  | 22 | exp Employee Absenteeism/ | 2393 |
|  |  |  | 23 | exp Job Performance/ | 22342 |
|  |  | Free text (ab,ti = title and abstract) | 24 | Wellbeing | 16417 |
|  |  |  | 25 | Well being | 91112 |
|  |  |  | 26 | Stress* | 278041 |
|  |  |  | 27 | Distress* | 80092 |
|  |  |  | 28 | Depress* | 325583 |
|  |  |  | 29 | Anxiet* | 212784 |
|  |  |  | 30 | Mental health | 199394 |
|  |  |  | 31 | Common mental disorder | 464 |
|  |  |  | 32 | Trauma* | 118083 |
|  |  |  | 33 | Resilienc* | 33001 |
|  |  |  | 34 | Post traumatic growth | 706 |
|  |  |  | 35 | Post-traumatic stress disorder | 11284 |
|  |  |  | 36 | PTSD | 35528 |
|  |  |  | 37 | Psychological* | 372933 |
|  |  |  | 38 | Coping | 81982 |
|  |  |  | 39 | Productivit* | 17816 |
|  |  |  | 40 | Efficien* | 90751 |
|  |  |  | 41 | Effectiveness* | 166463 |
|  |  |  | 42 | Output* | 29892 |
|  |  |  | 43 | Target* | 232686 |
|  |  |  | 44 | Work adj3 performanc* | 5613 |
|  |  |  | 45 | Works adj3 abilit* | 2970 |
|  |  |  | 46 | Work adj3 impair* | 914 |
|  |  |  | 47 | Work adj3 impact* | 4005 |
|  |  |  | 48 | Presenteeism | 585 |
|  |  | Combined Population / Context terms | 49 | 1 OR 2 OR 3 OR 4 OR 5 OR 6 OR 7 OR 8 OR 9 OR 10 OR 11 OR 12 OR 13 OR 14 | 12667 |
|  |  | Combined Outcome terms | 50 | 15 OR 16 OR 17 OR 18 OR 19 OR 20 OR 221 OR 22 OR 23 OR 24 OR 25 OR 26 OR 27 OR 28 OR 29 OR 30 OR 31 OR 32 OR 33 OR 34 OR 35 OR 36 OR 37 OR 38 OR 39 OR 40 OR 41 OR 42 OR 43 OR 44 OR 45 OR 46 OR 47 OR 48 | 2126661 |
|  |  | Final search string (1 + 2) | 51 | 49 AND 50 | 6142 |
|  |  | Final search string (1 + 2) Limited to 2020-Current | 52 | Limit 53 to yr="2020-Current" | **803** |

| **Concept** | **Combinations** | **Notes** | **Database: Ovid MEDLINE® ALL 1946 to January 25, 2022** | | |
| --- | --- | --- | --- | --- | --- |
|  |  |  | **Search Number** | **Terms** | **Results** |
| **(1) Population / Context (Homeworkers)** | **Combine with OR** | Controlled vocabulary | 1 | exp Teleworking/ | 182 |
|  |  | Free text (ab,ti = title and abstract) | 2 | (Work* adj3 home).ab,ti. | 7188 |
|  |  |  | 3 | WFH.ab,ti. | 256 |
|  |  |  | 4 | Flexible work*.ab,ti. | 773 |
|  |  |  | 5 | Virtual work*.ab,ti. | 298 |
|  |  |  | 6 | Mobile work*.ab,ti. | 151 |
|  |  |  | 7 | Home based work*.ab,ti. | 42 |
|  |  |  | 8 | Remote employe*.ab,ti. | 1 |
|  |  |  | 9 | E-work*.ab,ti. | 81 |
|  |  |  | 10 | Home work*.ab,ti. | 705 |
|  |  |  | 11 | Homework*.ab,ti. | 1878 |
|  |  |  | 12 | Remote work*.ab,ti. | 352 |
|  |  |  | 13 | Telecommut*.ab,ti. | 101 |
|  |  |  | 14 | Telework*.ab,ti. | 275 |
|  |  |  |  | **AND** |  |
| **(2) Outcomes (Mental health, resilience or productivity)** | **Combine with OR** | Controlled vocabulary | 15 | exp Work engagement/ | 717 |
|  |  |  | 16 | exp Work performance/ | 1123 |
|  |  |  | 17 | exp Mental health/ | 50241 |
|  |  |  | 18 | exp Posttraumatic growth/ | 402 |
|  |  |  | 19 | exp Resilience, Psychological/ | 7609 |
|  |  |  | 20 | exp Mental disorders/ | 1346697 |
|  |  | Free text (ab,ti = title and abstract) | 21 | Wellbeing.ab,ti. | 21817 |
|  |  |  | 22 | Well being.ab,ti. | 92614 |
|  |  |  | 23 | Stress*.ab,ti. | 946021 |
|  |  |  | 24 | Distress*.ab,ti. | 144311 |
|  |  |  | 25 | Depress*.ab,ti. | 507462 |
|  |  |  | 26 | Anxiet*.ab,ti. | 224856 |
|  |  |  | 27 | Mental health.ab,ti. | 174640 |
|  |  |  | 28 | Common mental disorder.ab,ti. | 711 |
|  |  |  | 29 | Trauma*.ab,ti. | 398686 |
|  |  |  | 30 | Resilienc*.ab,ti. | 35945 |
|  |  |  | 31 | Post traumatic growth.ab,ti. | 588 |
|  |  |  | 32 | Post-traumatic stress disorder.ab,ti. | 13237 |
|  |  |  | 33 | PTSD.ab,ti. | 28220 |
|  |  |  | 34 | Psychological*.ab,ti. | 247992 |
|  |  |  | 35 | Coping.ab,ti. | 62074 |
|  |  |  | 36 | Productivit*.ab,ti. | 69825 |
|  |  |  | 37 | Efficien*.ab,ti. | 1111935 |
|  |  |  | 38 | Effectiveness*.ab,ti. | 512710 |
|  |  |  | 39 | Output*.ab,ti. | 214785 |
|  |  |  | 40 | Target*.ab,ti. | 1761726 |
|  |  |  | 41 | Work adj 3 performanc*.ab,ti. | 6425 |
|  |  |  | 42 | Work adj3 abilit*.ab,ti. | 5692 |
|  |  |  | 43 | Work adj3 impair*.ab,ti. | 1934 |
|  |  |  | 44 | Work adj3 impact*.ab,ti. | 4514 |
|  |  |  | 45 | Presenteeism.ab,ti. | 1464 |
|  |  | Combined Population / Context terms | 46 | 1 OR 2 OR 3 OR 4 OR 5 OR 6 OR 7 OR 8 OR 9 OR 10 OR 11 OR 12 OR 13 OR 14 | 11093 |
|  |  | Combined Outcome terms | 47 | 15 OR 16 OR 17 OR 18 OR 19 OR 20 OR 21 OR 22 OR 23 OR 24 OR 25 OR 26 OR 27 OR 28 OR 29 OR 30 OR 31 OR 32 OR 33 OR 34 OR 35 OR 36 OR 37 OR 38 OR 39 OR 40 OR 41 OR 42 OR 43 OR 44 OR 45 | 6304179 |
|  |  | Final search string (1 + 2) | 48 | 46 AND 47 | 4830 |
|  |  | Final search string (1 + 2) Limited to 2020-Current | 49 | Limit | **1489** |

| **Concept** | **Combinations** | **Notes** | **Database: Ovid EMBASE** | | |
| --- | --- | --- | --- | --- | --- |
|  |  |  | **Search Number** | **Terms** | **Results** |
| **(1) Population / Context (Homeworkers)** | **Combine with OR** | Controlled vocabulary | 1 | exp Telecommuting/ | 952 |
|  |  |  | 2 | exp Work from home/ | 357 |
|  |  | Free text (ab,ti = title and abstract) | 3 | (Work* adj3 home).ab,ti. | 8996 |
|  |  |  | 4 | WFH.ab,ti. | 632 |
|  |  |  | 5 | Flexible work*.ab,ti. | 913 |
|  |  |  | 6 | Virtual work*.ab,ti. | 294 |
|  |  |  | 7 | Mobile work*.ab,ti. | 169 |
|  |  |  | 8 | Home based work*.ab,ti. | 45 |
|  |  |  | 9 | Remote employe*.ab,ti. | 3 |
|  |  |  | 10 | E-work*.ab,ti. | 103 |
|  |  |  | 11 | Home work*.ab,ti. | 937 |
|  |  |  | 12 | Homework*.ab,ti. | 2614 |
|  |  |  | 13 | Remote work*.ab,ti. | 362 |
|  |  |  | 14 | Telecommut*.ab,ti. | 90 |
|  |  |  | 15 | Telework*.ab,ti. | 250 |
|  |  |  |  | **AND** |  |
| **(2) Outcomes (Mental health, resilience or productivity)** | **Combine with OR** | Controlled vocabulary | 16 | exp psychological well-being/ | 23994 |
|  |  |  | 17 | exp Mental Health/ | 188761 |
|  |  |  | 18 | exp WellBeing/ | 100213 |
|  |  |  | 19 | exp Mental disease/ | 2386957 |
|  |  |  | 20 | exp Psychological resilience/ | 6529 |
|  |  |  | 21 | exp Job performance/ | 17413 |
|  |  |  | 22 | exp Productivity/ | 43874 |
|  |  | Free text (ab,ti = title and abstract) | 23 | Wellbeing.ab,ti. | 32722 |
|  |  |  | 24 | Well being.ab,ti. | 113373 |
|  |  |  | 25 | Stress*.ab,ti. | 1174559 |
|  |  |  | 26 | Distress*.ab,ti. | 200229 |
|  |  |  | 27 | Depress*.ab,ti. | 676213 |
|  |  |  | 28 | Anxiet*.ab,ti. | 317916 |
|  |  |  | 29 | Mental health.ab,ti. | 215572 |
|  |  |  | 30 | Common mental disorder.ab,ti. | 872 |
|  |  |  | 31 | Trauma*.ab,ti. | 507512 |
|  |  |  | 32 | Resilienc*.ab,ti. | 40908 |
|  |  |  | 33 | Post traumatic growth.ab,ti. | 802 |
|  |  |  | 34 | Post-traumatic stress disorder.ab,ti. | 17143 |
|  |  |  | 35 | PTSD.ab,ti. | 36523 |
|  |  |  | 36 | Psychological*.ab,ti. | 345868 |
|  |  |  | 37 | Coping.ab,ti. | 79864 |
|  |  |  | 38 | Productivit*.ab,ti. | 82222 |
|  |  |  | 39 | Efficien*.ab,ti. | 1298597 |
|  |  |  | 40 | Effectiveness*.ab,ti. | 683468 |
|  |  |  | 41 | Output*.ab,ti. | 253444 |
|  |  |  | 42 | Target*.ab,ti. | 2361781 |
|  |  |  | 43 | Work adj 3 performanc*.ab,ti. | 7618 |
|  |  |  | 44 | Work adj3 abilit*.ab,ti. | 7756 |
|  |  |  | 45 | Work adj3 impair*.ab,ti. | 3632 |
|  |  |  | 46 | Work adj3 impact.ab,ti. | 6172 |
|  |  |  | 47 | Presenteeism.ab,ti. | 2640 |
|  |  | Combined Population / Context terms | 48 | 1 OR 2 OR 3 OR 4 OR 5 OR 6 OR 7 OR 8 OR 9 OR 10 OR 11 OR 12 OR 13 OR 14 OR 15 | 8366364 |
|  |  | Combined Outcome terms | 49 | 16 OR 17 OR 18 OR 19 OR 20 OR 21 OR 22 OR 23 OR 24 OR 25 OR 26 OR 27 OR 28 OR 29 OR 30 OR 31 OR 32 OR 33 OR 34 OR 35 OR 36 OR 37 OR 38 OR 39 OR 40 OR 41 OR 42 OR 43 OR 44 OR 45 OR 46 OR 47 OR 48 | 14489 |
|  |  | Final search string (1 + 2) | 50 | 49 AND 50 | 6856 |
|  |  | Final search string (1 + 2) Limited to 2020-Current | 51 | 50 | 1777 |

| **Concept** | **Combinations** | **Notes** | **Database: Web of Science** | | |
| --- | --- | --- | --- | --- | --- |
|  |  |  | **Search Number** | **Terms** | **Results** |
| **(1) Population / Context (Homeworkers)** | **Combine with OR** |  | 1 | "Work from home" | 650 |
|  |  |  | 2 | "Work at home" | 435 |
|  |  |  | 3 | (Work* NEAR/3 "home") | 13681 |
|  |  |  | 4 | WFH | 381 |
|  |  |  | 5 | "Flexible work*" | 1211 |
|  |  |  | 6 | "Virtual work*" | 3830 |
|  |  |  | 7 | "Mobile work*" | 284 |
|  |  |  | 8 | "Home based work*" | 143 |
|  |  |  | 9 | "Remote employe*" | 6 |
|  |  |  | 10 | "E-work*" | 668 |
|  |  |  | 11 | "Home work*" | 862 |
|  |  |  | 12 | Homework* | 6375 |
|  |  |  | 13 | "Remote work*" | 616 |
|  |  |  | 14 | Telecommut* | 890 |
|  |  |  | 15 | Telework* | 1631 |
|  |  |  |  | **AND** |  |
| **(2) Outcomes (Mental health, resilience or productivity)** | **Combine with OR** |  | 16 | Wellbeing | 41439 |
|  |  |  | 17 | "Well being" | 145619 |
|  |  |  | 18 | Stress* | 2279141 |
|  |  |  | 19 | Distress* | 199277 |
|  |  |  | 20 | Depress* | 708191 |
|  |  |  | 21 | Anxiet* | 327766 |
|  |  |  | 22 | "Mental health" | 259891 |
|  |  |  | 23 | "Common mental disorder" | 817 |
|  |  |  | 24 | Trauma* | 425726 |
|  |  |  | 25 | Resilienc* | 127920 |
|  |  |  | 26 | "Post traumatic growth" | 1003 |
|  |  |  | 27 | "Post-traumatic stress disorder" | 15827 |
|  |  |  | 28 | PTSD | 40205 |
|  |  |  | 29 | Psychological* | 389340 |
|  |  |  | 30 | Coping | 101047 |
|  |  |  | 31 | Productivit* | 328389 |
|  |  |  | 32 | Efficien* | 3878989 |
|  |  |  | 33 | Effectiveness | 1154376 |
|  |  |  | 34 | Output* | 888717 |
|  |  |  | 35 | Target* | 2563812 |
|  |  |  | 36 | Work NEAR/3 "performanc*" | 48084 |
|  |  |  | 37 | Work NEAR/3 "abilit*" | 15738 |
|  |  |  | 38 | Work NEAR/3 "impair*" | 6487 |
|  |  |  | 39 | Work NEAR/3 "impact" | 21947 |
|  |  |  | 40 | Presenteeism | 2135 |
|  |  | Combined Population / Context terms | 41 | 1 OR 2 OR 3 OR 4 OR 5 OR 6 OR 7 OR 8 OR 9 OR 10 OR 11 OR 12 OR 13 OR 14 OR 15 | 28175 |
|  |  | Combined Outcome terms | 42 | 16 OR 17 OR 18 OR 19 OR 20 OR 21 OR 22 OR 23 OR 24 OR 25 OR 26 OR 27 OR 28 OR 29 OR 30 OR 31 OR 32 OR 33 OR 34 OR 35 OR 36 OR 37 OR 38 OR 39 OR 40 | 11583165 |
|  |  | Final search string (1 + 2) | 43 | 41 AND 42 | 10157 |
|  |  | Final search string (1 + 2) Limited to 2020-Current | 44 | 43 | 2837 |

**Supplemental Information Table 2: Grey literature Searches**

| **Search Engine** | **Search terms** | **Searched what?** | **Records found** |
| --- | --- | --- | --- |
| Ethos | Work from home AND wellbeing | Title, Abstract | 2 |
|  | Work from home AND mental health | Title, Abstract | 3 |
|  | Work from home AND productivity | Title, Abstract | 10 |
|  | Work from home AND resilience | Title, Abstract | 2 |
|  | Telework AND wellbeing | Title, Abstract | 1 |
|  | Telework AND mental health | Title, Abstract | 0 |
|  | Telework AND productivity | Title, Abstract | 3 |
|  | Telework AND resilience | Title, Abstract | 0 |
|  | ("work from home" OR telework OR homework) AND ("mental health" OR productivity OR resilience) | Any | 3 |
| Google Advanced search | ("work from home" OR telework OR homework) AND ("mental health" OR productivity OR resilience) | from Jan 2020-current, ordered by relevance | 17,400,000  (200 screened) |
| Gov.uk Publications | homework, telework, "work from home" | In: Research and Statistics or Policy Papers and Consultations | 78 |
| Office for National Statistics | homework, telework, "work from home" | General search | 45 |
| NHS Evidence | homework, telework, "work from home" | In: Primary research | 194 |
| **Total** |  |  | **541** |
